# Extra dural hematoma following a high voltage electrocution accident: A case report

**DOI:** 10.1016/j.amsu.2021.103157

**Published:** 2021-12-07

**Authors:** Marouane Makhchoune, Othman Benhayoun, Abdelkouddous Laaidi, Mohamed Yassine Haouas, Abdessamad Naja, Abdelhakim Lakhdar

**Affiliations:** a Neurosurgery Department, University Hospital Center IBN ROCHD, 1, Rue des Hôpitaux, Casablanca, Morocco; b Neurosurgery Department, University Hospital Center IBN ROCHD, Casablanca, Morocco

**Keywords:** Electrification, Hematoma, Complication, Surgery, Case report

## Abstract

Electrification accidents is a serious pathology due to the passage of an electric current through the body. The clinical manifestation is mainly represented by skin lesions with burners of different degrees however all the tissues of the body can be affected. The cerebral manifestation is a rare entity described in the literature. In this paper, we report the case of a patient with an electrocution accident manifested by an extradural hematoma who has not encountered a similar case described in the literature. We therefore present this case which poses a poorly understood pathophysiological problem.

## Highlights

• This paper presents a 44 years old man electrician was brought by his coworkers to emergency after he had an accident by electrocution to a high voltage current with direct cranial contact point at the vertex. The neurological examination revealed muscle power was 3/5 on right side, with 2nd and 3rd degree burns on his face and scalp.
• A cerebral CT scan was performed objectifying an extradural hematoma of the vertex with no sign of trauma the bone structure was intact. This one was surgically evacuated the follow-up was good at first but he developed septic shock which was the cause of his death.
• Cerebral bleeding due to electric shock are a rare entity described in the literature, their pathophysiological mechanism is still poorly understood, it is difficult to determine that these lesions are due to an accident of electric shock in real life or due to a head trauma associated.

## 1 Introduction

Electrification is defined as all the physiopathological manifestations related to the passage of current in the human body. Electrification accidents are due to direct or indirect contact of the body with a source of electric current with a difference in potential between 2 different points [1].

There are 2 types of electrification by high voltage currents and those by low voltage currents. Injury to the central nervous system due to electrification accidents is a rare occurrence, but nevertheless serious. In this paper we describe a case of accidental electrification by a high voltage current causing brain damage [1].

## 2 Case report

A 44 years old man electrician by profession, right-handed, with no particular pathological history. was brought by his coworkers to our emergency department after he had an accident at work by electrocution to a high voltage current with direct cranial contact point at the vertex, causing an initial loss of consciousness without associated head trauma. There is no history suggestive of any mental or physical illness.

**Fig. 1:**
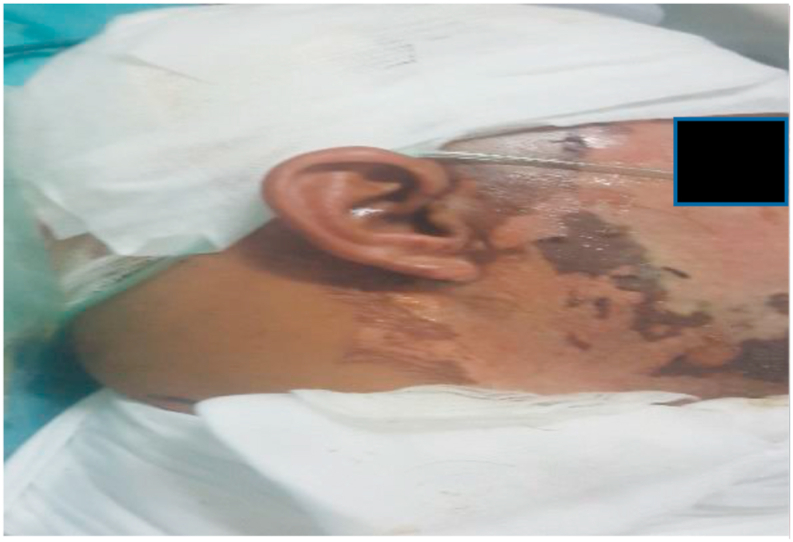
2nd and 3rd degree burns on the face.

**Fig. 2:**
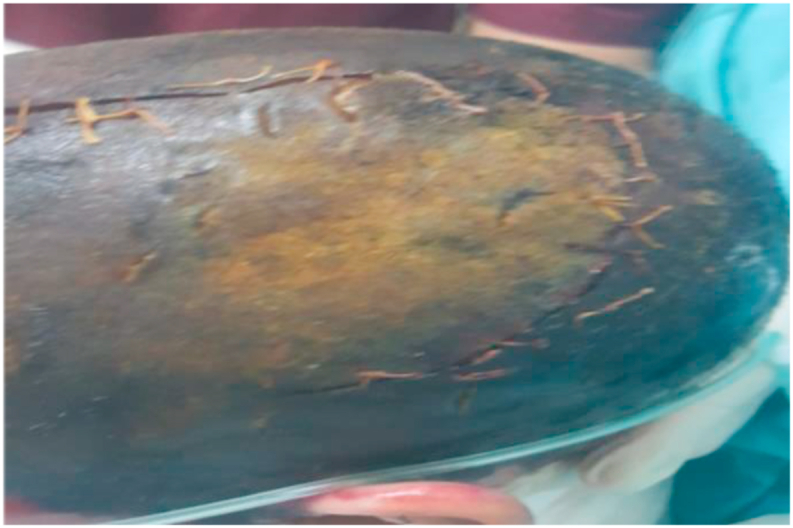
3rd degree burns in the scalp.

At admission, his GCS scores were E4V5M6. Neurological examination revealed muscle power was 3/5 on right side, 5/5 on left side, no sensitive deficiency with 2nd and 3rd degree burns on his face and scalp (Fig. 1, Fig. 2). The rest of the exam was normal.

**Fig. 3:**
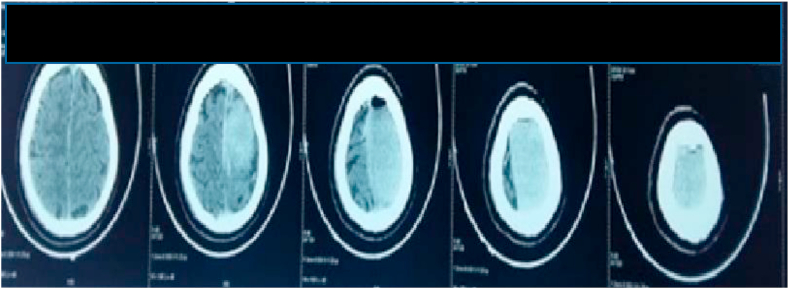
Extradural hematoma of the vertex axial cut.

**Fig. 4:**
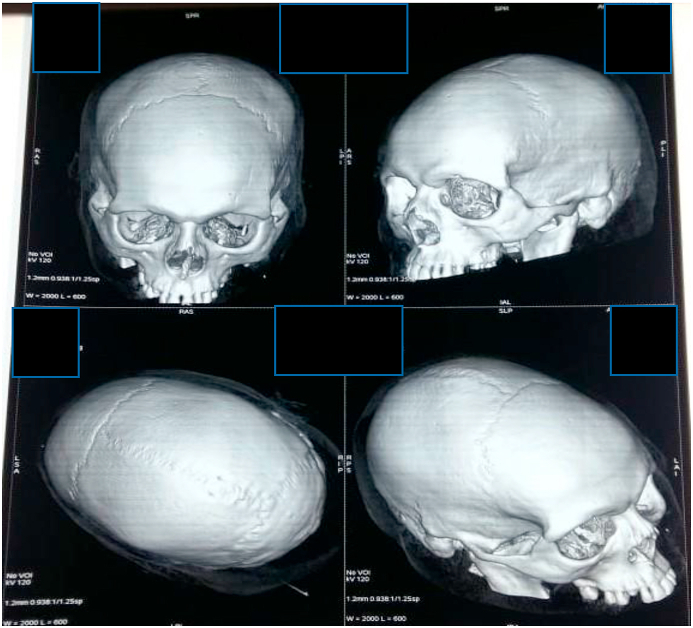
Bone reconstruction showing no traumatic lesions.

A cerebral CT scan was performed at the ER 4 hours after the accident objectifying an extradural hematoma of the vertex more marked on the left associated with a left frontal pneumocephaly there was no sign of trauma the bone structure was intact (Fig. 3, Fig. 4).

The diagnosis of extradural hematoma was obvious. We hospitalized the patient in the neurosurgical intensive care unit after being conditioned, the patient underwent a preoperative assessment, then was sent for surgical management. The surgery was performed under general anesthesia by our chief resident. After removal the bone flap we discovered an extradural hematoma which was evacuated, the origin of the bleeding was the superior longitudinal sinus there were no fracture in the bone.

**Fig. 5:**
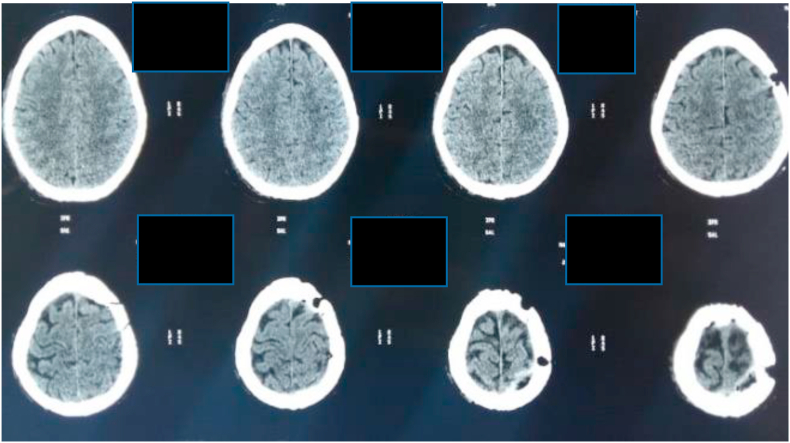
CT Scan post op showing a disappearance of the hematoma.

On the follow-up, there was total recovery from the motor deficiency, conscious; the control CT scan was satisfying (Fig. 5). The patient was later transferred to the burn center for additional care. the outcome was unfavorable the patient was in acute renal failure due to rhabdomyolysis associated with a lung infection unfortunately the patient developed septic shock which was the cause of his death.

This case has been reported in line with the 2020 SCARE guidelines [2].

## 3 Discussion

Brain injuries secondary to electrification accidents represent a rare entity in the literature. The major parts of clinical studies concerning the neurological manifestations of electrification accidents are made from post-lightning accidents. These complications can be central or peripheral, onset immediately or delayed, and transient or permanent [3].

The physiopathology mechanisms are linked to the direct or indirect effect of the electric current on any tissue of the organism that it encounters during its passage, knowing that the vessels and nerves offer the least resistance and are therefore the seat of the strongest intensity, from which it is recognized that the path of electrification is preferentially vascular-nervous. In addition to the electrothermal effect, there is a cellular effect which causes alterations in the plasma membrane with increased membrane permeability resulting in the formation of electropores in the membrane phospholipid layer [4]. These pores allow ions and certain intracellular molecules to pass through simple diffusion, and they have a limited lifespan. Besides these cellular effects, electricity has a specific role on the vessels, in particular on the arteries which cause thrombosis, weakening of the wall of small arteries which can breaking up causing bleeding [5]. This risk exists during the first three days following the initial accident, unlike aneurysms which may develop later. These small arteries are also the site of vasospasm due to dysregulation of the autonomic nervous system. We can also cite the anatomohistological data of post-lightning cerebral parenchymal involvement: diffuse congestion, edema, hemorrhage, swelling of lymphoid tissue. Histologically, there is vasodilation, hemorrhagic suffusions, degenerative endothelitis and sometimes hyperplasia with necrosis of the glial formations [[5], [6]].

Rougé and coll [6] propose, according to an experimental model, a histological classification of vascular lesions following electrification:

• stage 0: no vascular lesion;
• stage 1: small changes in the wall, often at the level of the intima, with edema or even detachment of endothelial cells, aggregation of blood along the wall; these lesions are localized in apparently healthy tissue in the vicinity of burned areas;
• stage II: more or less significant alterations of the vascular wall with necrosis, inflammatory infiltrate and thrombosis; these lesions are located in the peripheral areas of the burns;
• stage III: coagulation and necrosis of the entire vascular wall, located in the center of the burns.

Chaibdraa et al. [1]. In his article reports an observation of a 37-year-old patient without pathological history. Victim of accidental electric shock while handling an unprotected scrap bar that came into contact with a high voltage current with notion of projection and fall from its height. The examination found a conscious patient, well oriented in time and space, without motor deficit, hyperalgesic, a burnt body surface estimated at 26% associating deep lesions of degree IIa and IIb located at the level of the cephalic extremity (right hemiface), the anterior face of the neck, the right half trunk, right arm, and a preferential vasculo-nervous path mainly in the lower right limb. The patient presented on the 3rd day of his hospitalization with consciousness disorders associated with left pyramidal irritation [1]. The biological assessment was unremarkable.

**Fig. 6:**
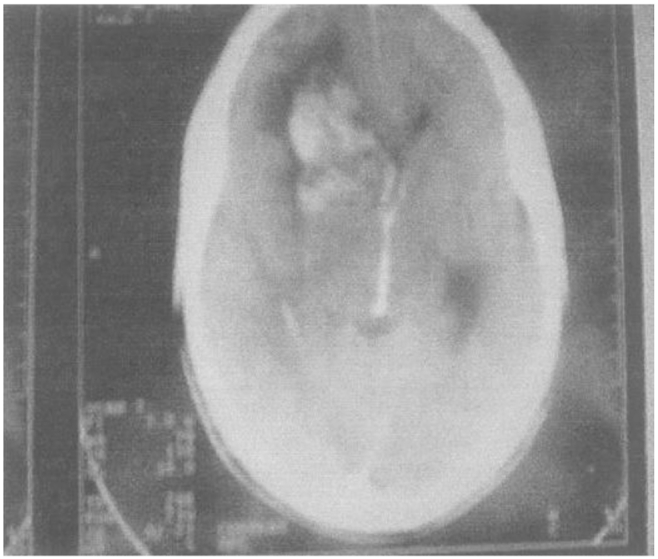
Right fronto basilar subarachnoid hemorrhage with ventricular flooding.

The brain CT scan showed subarachnoid bleeding (Fig. 6).

The conduct was stopping anticoagulants with neurological monitoring. The outcome was favorable with resorption as seen on the control CT scan, with resorption of the hematoma and intraventricular hemorrhage [[1], [8]].

The literature mentions cerebral hemorrhage especially in lightning strikes, but no such observation is reported in industrial current electrification, let alone extradural hematoma.

In the end, except in the presence of associated traumatic lesions, there does not seem to be a similar case described in the literature [7]. Electrification accident by contact with a high-voltage electric current carrying cable is considered to be the etiology of cerebral-meningeal hemorrhage on the following elements: exposure to high voltage current; initial brief loss of consciousness; the existence of lesions of cutaneous burns of the scalp testifying to the passage of the current by the ipsilateral cephalic end to the cerebral bleeding [[8], [9]].

## 4 Conclusion

Cerebral bleeding due to electric shock are a rare entity described in the literature, their pathophysiological mechanism is still poorly understood, it is difficult to determine that these lesions are due to an accident of electric shock in real life or due to a head trauma associated. A rigorous protection must be made as the helmet and the gloves to avoid this kind of work accident.

## Ethical approval

Written informed consent for publication of their clinical details and/or clinical images was obtained from the patient.

Ethical approval has been exempted by our institution.

## Sources of funding

None.

## Author contribution

Marouane MAKHCHOUNE: Corresponding author and writing the paper.

Othman BENHAYOUN: writing the paper.

Abdelkouddous LAAIDI: writing the paper.

Mohamed yassine HAOUAS: Correcting the paper.

Abdessamad NAJA: Correcting the paper.

Abdelhakim LAKHDAR: Correcting the paper.

## Research Registration Unique Identifying Number (UIN)

None.

## Guarantor

MAKHCHOUNE MAROUANE.

## Provenance and peer review

Provenance and peer review Not commissioned, externally peer-reviewed.

## Declaration of competing interest

The authors declare having no conflicts of interest for this article.
